# Supplementary material for: A Likelihood Approach for Real-Time Calibration of Stochastic Compartmental Epidemic Models
Source: PLoS Comput Biol. 2017 Jan 17;13(1):e1005257. doi: 10.1371/journal.pcbi.1005257 (PMC5240920; doi:10.1371/journal.pcbi.1005257)
Supplement: S1 File — (TAR.GZ) [file pcbi.1005257.s014.tar.gz › HSPH_Online-SI-Revision/PF08/n10-extreme/n10-extreme_PF_table4.pdf]

1	R0	{2.68692, 12.5884}	{2.92971, 19.8724}	{{2.88949, 20.}, {2.98967, 20.}}	{9.82136, 58.1626}	inf	669.	1729.77	{1696.9, 1784.72}	158.561	0.411142	newly	306.	536.25	{504., 565.}	75.2451	0.243142	Reff	0.305503	0.299472	{0.28349, 0.315716}	4.88346	ar	9089.	11 149.5	{11 020., 11 296.}	22.6704
2	R0	{1.79802, 12.5412}	{2.12386, 19.8357}	{{1.94214, 20.}, {2.2179, 20.}}	{18.2876, 58.1643}	inf	841.	1406.32	{1322.53, 1506.21}	67.2196	0.221536	newly	397.	463.8	{422., 496.}	16.927	0.0670096	Reff	0.656637	0.664801	{0.619325, 0.727929}	4.70791	ar	7412.	9564.31	{9168., 9784.}	29.0382
3	R0	{2.6174, 10.1105}	{2.97521, 17.931}	{{3., 17.7397}, {3., 18.2973}}	{13.6705, 77.3505}	inf	459.	1304.64	{1259.29, 1342.06}	184.234	0.45327	newly	256.	439.26	{414., 464.}	71.5859	0.234065	Reff	0.268022	0.253249	{0.244184, 0.256402}	7.61109	ar	9109.	10 742.6	{10 644., 10 840.}	17.9338
4	R0	{2.27532, 14.3149}	{2.13776, 19.2897}	{{2.05543, 17.2206}, {2.2209, 20.}}	{6.04572, 34.7528}	inf	750.	1226.85	{1014.51, 1370.79}	63.5795	0.210711	newly	320.	404.72	{364., 444.}	26.5	0.100799	Reff	0.473267	0.519783	{0.483125, 0.546339}	9.83201	ar	8478.	9542.28	{9289., 9728.}	12.5534
5	R0	{2.96649, 18.0674}	{2.82218, 19.8914}	{{2.76757, 20.}, {2.89713, 20.}}	{4.86457, 10.4421}	inf	1193.	1491.71	{1365.17, 1638.46}	25.2428	0.0967729	newly	386.	465.44	{419., 510.}	20.5907	0.0801015	Reff	0.251262	0.316832	{0.295562, 0.337394}	26.0965	ar	9476.	10 781.4	{10 620., 10 938.}	13.7756
6	R0	{2.94778, 16.0002}	{2.78308, 19.8522}	{{2.68404, 20.}, {2.85999, 20.}}	{5.71628, 24.0746}	inf	1090.	1758.25	{1588.06, 1921.52}	61.3076	0.20653	newly	362.	552.	{494., 594.}	52.4862	0.182087	Reff	0.295957	0.377303	{0.353369, 0.411856}	27.4857	ar	9321.	10 652.7	{10 486., 10 779.}	14.2874
7	R0	{2.17562, 11.7184}	{2.67973, 18.8488}	{{2.27862, 15.561}, {2.9529, 20.}}	{23.2075, 60.8477}	inf	793.	1676.61	{1337.86, 1849.29}	111.427	0.320616	newly	406.	553.79	{500., 606.}	36.4015	0.133656	Reff	0.496041	0.403712	{0.348166, 0.482165}	18.966	ar	8345.	10 374.6	{9899., 10 642.}	24.321
8	R0	{2.7904, 17.8966}	{2.33678, 19.5203}	{{2.12446, 17.3521}, {2.45102, 20.}}	{16.2563, 10.2124}	inf	1403.	1530.9	{1341.57, 1615.05}	10.7125	0.0441018	newly	475.	498.27	{465., 528.}	6.00632	0.0251337	Reff	0.341266	0.487235	{0.453935, 0.543593}	42.7728	ar	9285.	10 090.2	{9653., 10 281.}	8.67205
9	R0	{2.51574, 16.376}	{2.44258, 19.8769}	{{2.26784, 20.}, {2.52994, 20.}}	{4.39723, 21.3781}	inf	1116.	1512.25	{1428.5, 1588.8}	35.5061	0.131544	newly	427.	479.4	{444., 517.}	12.7494	0.0515186	Reff	0.397235	0.430558	{0.392542, 0.491254}	11.0028	ar	8937.	10 345.2	{10 164., 10 532.}	15.7565
10	R0	{2.56405, 18.1787}	{2.23053, 18.5235}	{{2.13049, 17.5306}, {2.41378, 20.}}	{13.0253, 4.25287}	inf	1437.	1505.2	{1380.04, 1630.23}	6.66993	0.0275042	newly	467.	518.59	{477., 558.}	11.2612	0.0456007	Reff	0.441786	0.561891	{0.519065, 0.608345}	27.1872	ar	9005.	10 221.3	{9951., 10 598.}	13.5069
11	R0	{2.475, 17.1709}	{1.95079, 20.}	{{1.92692, 20.}, {1.97565, 20.}}	{21.1803, 16.4761}	inf	1270.	1316.79	{1295.67, 1340.76}	3.72896	0.0158599	newly	447.	430.08	{404., 454.}	4.61745	0.0207331	Reff	0.449708	0.651875	{0.636809, 0.664072}	44.9554	ar	8870.	9450.47	{9333., 9557.}	6.54419
12	R0	{2.14974, 11.8619}	{2.23663, 18.7269}	{{2.09645, 17.477}, {2.45132, 20.}}	{5.01807, 57.874}	inf	994.	1567.99	{1426.19, 1661.3}	57.7455	0.197114	newly	494.	534.24	{505., 562.}	8.23887	0.033962	Reff	0.487776	0.55951	{0.475816, 0.607973}	15.8431	ar	8414.	10 012.8	{9705., 10 352.}	19.0018
13	R0	{2.20385, 14.4767}	{2.17248, 20.}	{{2.00168, 20.}, {2.25333, 20.}}	{3.09931, 38.153}	inf	760.	1150.12	{1085.26, 1245.14}	51.3319	0.17933	newly	319.	371.69	{340., 411.}	16.5235	0.0651959	Reff	0.478235	0.560776	{0.524261, 0.615194}	17.2593	ar	8347.	9788.56	{9577., 9973.}	17.2704
14	R0	{2.35671, 13.8357}	{2.55884, 18.9509}	{{2.12928, 14.6731}, {2.80954, 20.}}	{11.8967, 37.4587}	inf	848.	1385.16	{905.815, 1596.25}	64.0367	0.208451	newly	361.	450.92	{381., 508.}	25.4127	0.0962187	Reff	0.411717	0.388911	{0.313509, 0.473398}	15.41	ar	8719.	10 111.8	{9508., 10 428.}	16.021
15	R0	{1.91368, 10.1082}	{2.16304, 19.4406}	{{2.08591, 18.0009}, {2.20736, 20.}}	{13.0302, 92.3248}	inf	436.	1317.14	{1150.88, 1434.57}	202.096	0.478576	newly	261.	433.75	{398., 467.}	66.1877	0.219854	Reff	0.581759	0.537858	{0.521846, 0.555377}	7.86842	ar	7486.	9762.52	{9562., 9907.}	30.4104
16	R0	{2.48727, 14.882}	{2.72396, 20.}	{{2.50258, 20.}, {2.87003, 20.}}	{9.80688, 34.3905}	inf	1044.	1800.43	{1734.15, 1889.55}	72.4552	0.236406	newly	425.	559.55	{517., 596.}	31.6588	0.118743	Reff	0.402192	0.366557	{0.316873, 0.438783}	14.3097	ar	8894.	11 011.	{10 826., 11 168.}	23.8021
17	R0	{2.46795, 13.1915}	{2.86306, 19.3874}	{{2.54458, 17.7908}, {3., 20.}}	{16.7031, 46.9691}	inf	950.	1645.59	{1304.34, 1806.38}	73.2195	0.236113	newly	457.	523.53	{454., 572.}	15.1969	0.0604197	Reff	0.428189	0.336664	{0.284299, 0.407966}	21.9765	ar	8796.	10 588.1	{10 293., 10 757.}	20.374
18	R0	{2.96603, 16.7543}	{2.81199, 19.9937}	{{2.77057, 20.}, {2.86709, 20.}}	{5.1936, 19.3346}	inf	1079.	1526.68	{1454.45, 1601.98}	41.4899	0.150334	newly	402.	469.42	{437., 499.}	16.7711	0.0666718	Reff	0.284146	0.316474	{0.298861, 0.328324}	11.3775	ar	9394.	10 681.7	{10 576., 10 800.}	13.7079
19	R0	{2.25216, 9.90465}	{2.94453, 19.7889}	{{2.844, 20.}, {3., 20.}}	{30.7426, 99.7939}	inf	643.	1911.75	{1618.21, 2046.98}	197.317	0.471643	newly	379.	596.42	{550., 665.}	57.3668	0.195551	Reff	0.403812	0.31396	{0.281606, 0.368109}	22.7827	ar	8631.	11 425.	{11 251., 11 619.}	32.3719
20	R0	{2.70837, 18.1923}	{2.44871, 19.7793}	{{2.25419, 18.5721}, {2.56062, 20.}}	{9.58714, 9.24603}	inf	1399.	1648.1	{1568.94, 1728.61}	17.8053	0.070726	newly	468.	529.69	{492., 568.}	13.3184	0.0537628	Reff	0.365088	0.471469	{0.42066, 0.542125}	29.1382	ar	9183.	10 491.9	{10 196., 10 670.}	14.253
21	R0	{2.30288, 15.5877}	{2.21087, 20.}	{{2.17091, 20.}, {2.25007, 20.}}	{3.99539, 28.3063}	inf	1029.	1324.13	{1224.16, 1376.46}	28.681	0.109064	newly	405.	429.49	{390., 460.}	7.50864	0.0311724	Reff	0.441923	0.5882	{0.559788, 0.609381}	33.1001	ar	8674.	9783.99	{9637., 9890.}	12.7967
22	R0	{2.82208, 10.7544}	{2.65987, 15.9328}	{{2.61789, 15.3426}, {2.69709, 16.4955}}	{6.2524, 48.1515}	inf	586.	1298.89	{1211.83, 1406.85}	121.654	0.344091	newly	278.	492.39	{457., 524.}	77.1187	0.247553	Reff	0.244392	0.345499	{0.339904, 0.353391}	41.3708	ar	9333.	10 294.3	{10 210., 10 382.}	10.2999
23	R0	{1.69923, 10.1366}	{2.11244, 18.9906}	{{1.8516, 16.8787}, {2.33806, 20.}}	{24.3175, 87.3471}	inf	558.	1373.73	{1173.21, 1629.46}	146.188	0.388447	newly	330.	466.1	{412., 526.}	41.2424	0.148083	Reff	0.599318	0.61275	{0.539884, 0.716869}	9.33127	ar	7055.	9787.77	{9164., 10 218.}	38.7352
24	R0	{2.64944, 19.054}	{2.17307, 19.9039}	{{2.13978, 20.}, {2.19677, 20.}}	{17.9802, 5.07191}	inf	1099.	1195.03	{1134.85, 1250.51}	10.1334	0.0421377	newly	382.	381.86	{344., 408.}	5.0733	0.0223008	Reff	0.35317	0.495395	{0.483059, 0.505546}	40.2708	ar	9147.	9857.22	{9744., 9965.}	7.76451
25	R0	{2.53191, 10.9214}	{2.47148, 19.9695}	{{2.4334, 20.}, {2.49922, 20.}}	{2.38681, 82.8472}	inf	571.	1542.1	{1490.97, 1604.6}	170.069	0.431273	newly	309.	487.44	{459., 517.}	57.7476	0.197451	Reff	0.310159	0.440475	{0.426121, 0.459971}	42.0159	ar	9009.	10 108.8	{10 030., 10 193.}	12.2079
26	R0	{1.89155, 6.76944}	{2.88715, 18.2856}	{{2.59477, 15.892}, {3., 20.}}	{52.6342, 170.12}	inf	234.	1332.52	{1120.89, 1628.68}	469.451	0.750742	newly	188.	443.04	{396., 503.}	135.66	0.370684	Reff	0.540416	0.317868	{0.279753, 0.364577}	41.1809	ar	7348.	10 475.2	{10 257., 10 663.}	42.5591
27	R0	{2.63149, 15.6979}	{2.67209, 20.}	{{2.5313, 20.}, {2.8208, 20.}}	{4.59588, 27.4054}	inf	1228.	1764.95	{1715.49, 1835.83}	43.7257	0.157344	newly	463.	551.39	{520., 580.}	19.0907	0.07541	Reff	0.367882	0.386458	{0.342229, 0.435275}	10.3993	ar	9104.	10 866.6	{10 738., 10 992.}	19.3607
28	R0	{2.85141, 12.6992}	{2.83216, 17.3379}	{{2.70408, 16.0676}, {3., 20.}}	{3.80185, 36.5278}	inf	776.	1394.06	{1237.29, 1717.75}	79.6463	0.251207	newly	347.	484.63	{450., 525.}	39.6628	0.144312	Reff	0.251494	0.293845	{0.268366, 0.323802}	16.8395	ar	9317.	10 711.4	{10 547., 10 899.}	14.9661
29	R0	{2.54244, 12.9085}	{2.12322, 18.2043}	{{2.05511, 17.4232}, {2.21744, 20.}}	{16.4888, 41.0254}	inf	779.	1287.93	{1170.74, 1510.7}	65.3315	0.216303	newly	343.	450.24	{419., 488.}	31.2653	0.117384	Reff	0.329754	0.542817	{0.524144, 0.573899}	64.6123	ar	9034.	9621.69	{9390., 9908.}	6.50531
30	R0	{2.10982, 11.7636}	{2.14955, 18.7527}	{{2.08488, 17.5021}, {2.25636, 20.}}	{2.61692, 59.4133}	inf	703.	1404.16	{1249.07, 1659.61}	99.7389	0.298458	newly	366.	478.05	{430., 532.}	30.6148	0.114528	Reff	0.486735	0.559089	{0.51016, 0.596401}	14.8652	ar	8224.	9907.68	{9679., 10 177.}	20.4728
31	R0	{2.76312, 15.5396}	{2.70186, 18.7693}	{{2.34886, 15.1395}, {3., 20.}}	{6.67837, 22.1577}	inf	1113.	1455.12	{1066.99, 1724.53}	32.9486	0.120988	newly	439.	474.66	{422., 534.}	10.1913	0.0414064	Reff	0.324667	0.350381	{0.265563, 0.413502}	13.3109	ar	9220.	10 420.9	{10 009., 10 704.}	13.0252
32	R0	{2.21727, 15.5439}	{1.82131, 20.}	{{1.77922, 20.}, {1.93025, 20.}}	{17.8581, 28.6678}	inf	936.	1117.66	{1013.56, 1165.29}	19.4081	0.0765442	newly	399.	367.64	{335., 398.}	8.29073	0.0383069	Reff	0.506203	0.688329	{0.601716, 0.718195}	35.9788	ar	8359.	9325.16	{9117., 9632.}	11.5583
33	R0	{2.72912, 10.8159}	{3., 20.}	{{3., 20.}, {3., 20.}}	{9.92554, 84.913}	inf	395.	1378.32	{1339.38, 1435.97}	248.942	0.54256	newly	210.	420.2	{398., 443.}	100.095	0.300826	Reff	0.252989	0.23462	{0.227879, 0.24199}	7.26078	ar	9196.	11 140.1	{11 049., 11 236.}	21.1404
34	R0	{2.20928, 10.1308}	{2.908, 18.4331}	{{2.41514, 12.8212}, {3., 20.}}	{31.6265, 81.9513}	inf	446.	1381.04	{734.108, 1593.3}	209.651	0.478278	newly	238.	448.02	{345., 502.}	88.2437	0.271297	Reff	0.406066	0.269844	{0.243597, 0.323166}	33.5467	ar	8388.	10 577.	{9997., 10 742.}	26.097
35	R0	{2.10103, 15.2673}	{2.14385, 18.7889}	{{2.05006, 17.2195}, {2.20386, 20.}}	{3.44464, 23.2228}	inf	986.	1308.14	{1128.48, 1479.93}	33.261	0.122879	newly	382.	443.97	{399., 490.}	16.7147	0.0660811	Reff	0.508869	0.543095	{0.506412, 0.592619}	7.8089	ar	8264.	9704.89	{9448., 9901.}	17.4357
36	R0	{2.74914, 19.2775}	{2.24108, 20.}	{{2.18934, 20.}, {2.28113, 20.}}	{18.4808, 3.74789}	inf	1441.	1353.69	{1300.64, 1411.99}	6.224	0.0282219	newly	436.	435.08	{407., 463.}	4.09633	0.0178092	Reff	0.366735	0.556105	{0.535754, 0.567627}	51.6367	ar	9301.	10 065.9	{9951., 10 174.}	8.22363
37	R0	{2.78161, 16.0136}	{2.61616, 19.9885}	{{2.47803, 20.}, {2.80224, 20.}}	{6.34906, 24.822}	inf	1033.	1532.57	{1387.21, 1681.35}	48.3607	0.17016	newly	388.	477.84	{423., 534.}	23.1546	0.08881	Reff	0.3146	0.381765	{0.314556, 0.427613}	21.424	ar	9240.	10 713.9	{10 544., 10 879.}	15.9516
38	R0	{2.4454, 8.33501}	{3., 20.}	{{3., 20.}, {3., 20.}}	{22.6793, 139.952}	inf	243.	1765.13	{1698.08, 1818.71}	626.392	0.861047	newly	147.	538.13	{512., 559.}	266.075	0.563268	Reff	0.306653	0.254367	{0.250417, 0.258734}	17.0505	ar	8853.	11 538.1	{11 362., 11 670.}	30.3293
39	R0	{2.9154, 12.3861}	{2.96201, 19.5709}	{{2.77083, 17.406}, {3., 20.}}	{3.41332, 58.0066}	inf	596.	1484.45	{1297.78, 1575.69}	149.069	0.394046	newly	284.	463.99	{428., 495.}	63.3768	0.212216	Reff	0.225069	0.278591	{0.2667, 0.304924}	23.7805	ar	9371.	10 505.2	{10 351., 10 604.}	12.1032
40	R0	{2.40923, 15.1963}	{2.40165, 19.6214}	{{2.19459, 17.8396}, {2.56344, 20.}}	{3.95228, 29.1195}	inf	1025.	1548.71	{1425.1, 1654.27}	51.0939	0.178579	newly	421.	500.84	{460., 538.}	18.9644	0.0747526	Reff	0.424988	0.478638	{0.415689, 0.534942}	13.6794	ar	8735.	10 536.6	{10 125., 10 778.}	20.6247
41	R0	{2.63137, 15.238}	{2.24541, 18.6037}	{{2.14296, 17.5526}, {2.45031, 20.}}	{14.6676, 22.0874}	inf	1020.	1269.67	{1149.48, 1403.92}	24.4777	0.0938143	newly	409.	429.91	{398., 464.}	6.41809	0.026672	Reff	0.336026	0.496915	{0.434269, 0.524453}	47.8799	ar	9150.	9955.77	{9722., 10 259.}	8.80623
42	R0	{2.48769, 19.4593}	{1.9807, 20.}	{{1.94707, 20.}, {2.0097, 20.}}	{20.3798, 2.77862}	inf	1184.	1037.36	{997.891, 1066.95}	12.3848	0.057665	newly	394.	334.7	{315., 359.}	15.0508	0.0714175	Reff	0.413205	0.57774	{0.556346, 0.597706}	39.8191	ar	8932.	9788.04	{9630., 9913.}	9.58397
43	R0	{2.61199, 10.214}	{2.96614, 19.6267}	{{2.76878, 17.6137}, {3., 20.}}	{13.5699, 92.1545}	inf	551.	1864.35	{1672.67, 1986.63}	238.358	0.527775	newly	296.	586.51	{531., 628.}	98.1453	0.296193	Reff	0.299073	0.329728	{0.302789, 0.358514}	10.2502	ar	9061.	10 504.8	{10 350., 10 605.}	15.934
44	R0	{2.50994, 18.3382}	{1.92951, 20.}	{{1.90699, 20.}, {1.9461, 20.}}	{23.1251, 9.06196}	inf	1257.	1274.24	{1252., 1310.85}	2.50697	0.0108961	newly	414.	416.67	{388., 441.}	4.1087	0.0178855	Reff	0.47714	0.650466	{0.634248, 0.672038}	36.3261	ar	8827.	9323.56	{9187., 9443.}	5.62547
45	R0	{2.44283, 16.8338}	{2.23987, 19.1374}	{{2.10054, 17.5214}, {2.3627, 20.}}	{8.30838, 13.6845}	inf	1098.	1393.95	{1221.2, 1523.41}	26.9533	0.102047	newly	418.	463.29	{425., 498.}	11.012	0.0444451	Reff	0.427251	0.494963	{0.448554, 0.534764}	15.8482	ar	8878.	10 182.7	{9886., 10 435.}	14.6953
46	R0	{2.51815, 18.1462}	{2.22189, 20.}	{{2.06448, 20.}, {2.27716, 20.}}	{11.7649, 10.2159}	inf	1305.	1322.56	{1216.7, 1390.33}	4.80589	0.0207235	newly	464.	427.73	{387., 466.}	8.55819	0.0397107	Reff	0.458051	0.562529	{0.473903, 0.596378}	22.8092	ar	8978.	9967.06	{9790., 10 123.}	11.0165
47	R0	{2.88885, 16.8987}	{2.13321, 19.0035}	{{2.04834, 17.4558}, {2.20861, 20.}}	{26.1571, 12.4553}	inf	1290.	1473.12	{1267.77, 1667.8}	14.9543	0.0582594	newly	468.	496.45	{458., 543.}	7.46368	0.030869	Reff	0.329618	0.583497	{0.550319, 0.603578}	77.0223	ar	9337.	9654.07	{9383., 9854.}	3.41684
48	R0	{2.58709, 13.8786}	{2.87494, 19.9968}	{{2.84156, 20.}, {2.91589, 20.}}	{11.1265, 44.0836}	inf	1037.	1797.21	{1665.61, 1924.48}	73.3082	0.238211	newly	435.	557.23	{506., 607.}	28.0989	0.106415	Reff	0.330113	0.330322	{0.317417, 0.350232}	3.2542	ar	9146.	11 020.5	{10 912., 11 098.}	20.495
49	R0	{2.42959, 10.7132}	{3., 20.}	{{3., 20.}, {3., 20.}}	{23.4776, 86.6856}	inf	676.	1749.16	{1711.9, 1802.72}	158.751	0.412785	newly	359.	534.59	{504., 567.}	48.9109	0.172482	Reff	0.358122	0.273512	{0.26441, 0.286603}	23.626	ar	8863.	10 984.9	{10 864., 11 108.}	23.9407
50	R0	{2.60513, 13.2796}	{2.76421, 20.}	{{2.71826, 20.}, {2.79315, 20.}}	{6.16197, 50.6069}	inf	1051.	1743.76	{1718.86, 1765.22}	65.9139	0.219847	newly	465.	540.76	{514., 568.}	16.2925	0.0652083	Reff	0.354037	0.382173	{0.372728, 0.389919}	7.94724	ar	9095.	10 606.8	{10 482., 10 727.}	16.6228
